# Anti-inflammatory cytokine-eluting collagen hydrogel reduces the host immune response to dopaminergic cell transplants in a rat model of Parkinson’s disease

**DOI:** 10.1042/NS20210028

**Published:** 2021-08-23

**Authors:** Sílvia Cabré, Verónica Alamilla, Niamh Moriarty, Abhay Pandit, Eilís Dowd

**Affiliations:** 1 Pharmacology and Therapeutics and Galway Neuroscience Centre, National University of Ireland (NUI), Galway, Ireland; 2 CÚRAM SFI Research Centre for Medical Devices, National University of Ireland (NUI), Galway, Ireland; 3 Anatomy and Neuroscience and APC Microbiome Institute, University College Cork (UCC), Cork, Ireland; 4 The Florey Institute of Neuroscience and Mental Health, The University of Melbourne, Parkville 3052, Victoria, Australia

**Keywords:** biomaterials, cell transplantation, collagen hydrogels, IL-10, microglia, Parkinson's disease

## Abstract

In cell replacement approaches for Parkinson’s disease, the intracerebral implantation of dopamine neuron-rich grafts generates a neuroinflammatory response to the grafted cells that contributes to its varied outcome. Thus, the aim of the present study was to fabricate an anti-inflammatory cytokine-eluting collagen hydrogel capable of delivering interleukin (IL)-10 to the brain for reduction of the neuroinflammatory response to intracerebral cellular grafts. *In vitro* assessment revealed that cross-linker concentration affected the microstructure and gelation kinetics of the hydrogels and their IL-10 elution kinetics, but not their cytocompatibility or the functionality of the eluted IL-10. *In vivo* evaluation revealed that the hydrogels were capable of delivering and retaining IL-10 in the rat striatum, and reducing the neuroinflammatory (microglial) response to hydrogel-encapsulated grafts. In conclusion, IL-10-eluting collagen hydrogels may have beneficial anti-inflammatory effects in the context of cellular brain repair therapies for Parkinson’s disease and should be investigated further.

## Background

Cell transplantation is a promising disease-modifying therapy that could develop into an alternative treatment for Parkinson’s disease [1]. However, a significant limitation of this therapy that has prevented its progression into the clinic is its variable outcome which is associated with multiple factors including the host brain’s inflammatory response to the cellular implant [2]. It is well known that the transplantation of exogenous cells into the brain generates an innate inflammatory response [3–9] and that only a small portion (1–20%) of the grafted dopaminergic neurons from fetal midbrain tissue survive the transplantation process [10]. In the first instance, when cells are transplanted into the brain, a gliotic reaction is initiated, with the recruitment of microglia to the vicinity of the graft site, alongside the recruitment of astrocytes to seal off the traumatic injury caused by the implant. Thus, the host innate immune response is characterised by the recruitment of microglia and astrocytes to the grafted site and surroundings. This host innate immune response is very quick [6,9] – present within hours after injection – and contributes to the variable reparative outcome of such transplantation approaches [3,5]. Thus, there is a clear window to explore the potential benefits of targeting the host innate immune response at the grafted site to challenge the extensive cell death of dopaminergic neurons within the first stages after transplantation. However, despite this, the effects of targeting the host innate immune response locally at the site of cell transplantation to enhance cell survival during the early transplantation phase have been poorly investigated.

In recent years, biomaterial scaffolds have been investigated in an attempt to reduce or overcome the current challenges surrounding the cell transplantation processes [2,11]. In particular, chemically cross-linked collagen-based scaffolds have been broadly used in the tissue engineering field due to collagen’s low immunogenicity, biodegradability, high availability and versatility [12]. In Parkinson’s disease research, these naturally derived, *in situ*-forming, injectable scaffolds have been proven to improve the survival, re-innervation and functional capability of intrastriatal transplanted fetal dopaminergic neurons when used alone or in conjunction with the neurotrophin, glial cell line-derived neurotrophic factor (GDNF), in a rodent model of Parkinson’s disease [13,14]. Additionally, these collagen scaffolds are capable of substantially reducing the innate inflammatory response around the grafted cells by creating a physical barrier between the transplanted and host cells [14,15]. Since these hydrogels have the capacity to retain and release therapeutic factors, it should be possible to further enhance their protective effect by the incorporation of anti-inflammatory molecules, such as the anti-inflammatory cytokine, interleukin (IL)-10 (IL-10), which is well known to suppress microglial activation [16].

**Figure 1:**
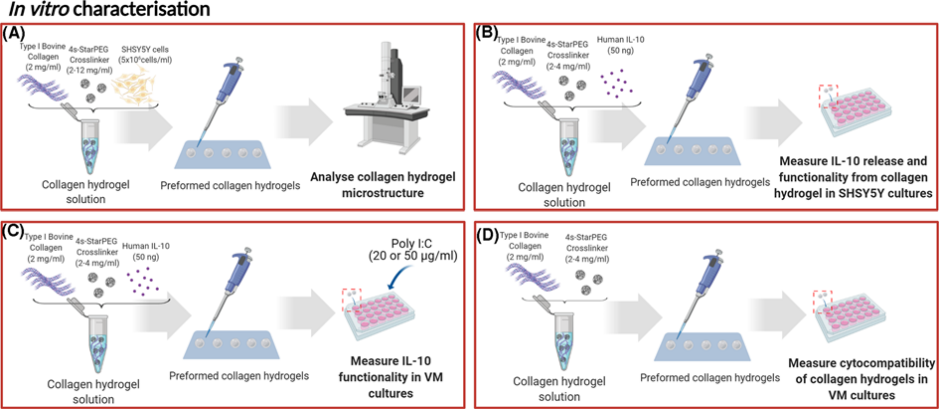
Preliminary *in vitro* experimental designs Before progressing to *in vivo* studies, a series of *in vitro* studies were completed to characterise the IL-10-eluting collagen hydrogel. Specifically, (**A**) SEM was used to visualise the hydrogel microstructure alone or when encapsulating SH-SY5Y cells, (**B**) the IL-10 elution kinetics from the hydrogel was assessed in SH-SY5Y cell cultures, (**C**) the anti-inflammatory functionality and the (**D**) cytocompatibility of the IL-10-eluting hydrogel was assessed in primary neural cultures. Abbreviation: SEM, scanning electron microscopy.

**Figure 2:**
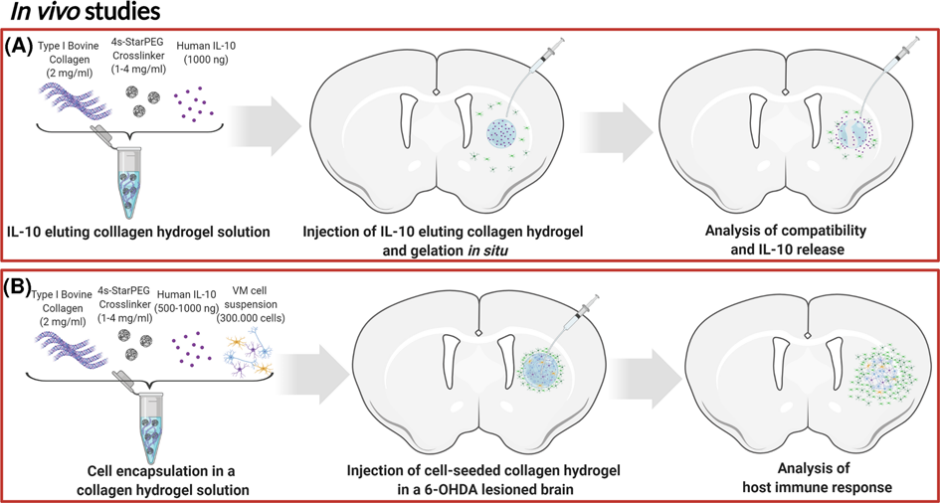
*In vivo* experimental designs Following *in vitro* characterisation, two *in vivo* studies were completed to assess (**A**) the neurocompatibility of the IL-10-eluting hydrogel and its ability to deliver and retain IL-10 in the striatum in naïve rats, and to assess (**B**) the ability of the IL-10-eluting hydrogel to reduce the host inflammatory response to primary fetal dopamine neuron-rich transplants.

Therefore, the present study aimed to generate and characterise an injectable collagen scaffold in an injectable hydrogel form capable of delivering functional IL-10 to the brain to reduce the inflammatory response to dopamine neuron-rich grafts in the context of cell-based brain repair for Parkinson’s disease. To do so, we generated several hydrogel compositions using different concentrations of cross-linker and assessed these *in vitro* for their microstructure and gelation kinetics, as well as their IL-10 elution kinetics, anti-inflammatory functionality and cytocompatibility (Figure 1). After this initial characterisation, we assessed the IL-10-eluting hydrogels *in vivo* in terms of their ability to deliver and retain IL-10 in the striatum and their ability to reduce the neuroinflammatory response to dopaminergic grafts (Figure 2).

## Methods

### *In vitro* studies

#### Fabrication of type I bovine collagen hydrogels

All components were maintained on ice to prevent premature gelation. For a final volume of 1000 µl, 400 µl of 5 mg/ml type I bovine collagen (Vornia Biomaterials) stock solution, neutralised with 1 M NaOH, was added to 200 µl of 10× phosphate-buffered saline (PBS) containing the required concentration of the cross-linker, poly(ethylene glycol) ether tetrasuccinimidyl glutarate (4s-StarPEG). The volume was made up to 1000 μl by adding 400 μl of PBS alone or, for the IL-10-eluting hydrogels, PBS containing the required concentration of human IL-10 (GFH83-100, Cell Guidance Systems). Thus, the resulting collagen hydrogel had a final concentration of 2 mg/ml of type I bovine collagen. Based on our previous studies using collagen hydrogels for release of the neurotrophin, GDNF [13,14], we focused primarily on hydrogels cross-linked with 1, 2 and/or 4 mg/mg cross-linker for the IL-10 eluting hydrogels *in vitro* and *in vivo*. Some additional concentrations were included for the preliminary visualisation and gelation assays. Hydrogels with all cross-linker concentrations were injectable as, because there were kept on ice before injection, they remained in their liquid state and could easily pass through the injection cannula.

#### Visualisation of the collagen hydrogel microstructure

To visualise the differently cross-linked collagen hydrogels, 200 µl collagen hydrogels with increasing cross-linker concentrations (2–12 mg/ml) were generated and frozen immediately in liquid nitrogen. Once frozen, collagen hydrogels were fractured (to allow for visualisation of the inside of the sample) and freeze-dried overnight. Samples were placed into aluminium stubs and gold-coated in a gold sputter coater Emitech K550 (Quorumtech, U.K.) or Emscope SC500 (Quorumtech, U.K.). Images from the sample’s structure were taken using a Hitachi S-4700N Pressure Scanning Electron Microscope (Hitachi, U.K.). To determine the impact of cross-linker concentration on porosity of the hydrogels, pore diameter measurements were taken from the scanning electron microscopy (SEM) photomicrographs using ImageJ software (20 randomly selected pores per hydrogel; 3 hydrogels per concentration).

To observe the distribution of the cells within the hydrogel, SH-SY5Y cells (ATCC; 5 × 10^6^ cells/ml) were encapsulated in 50 μl collagen hydrogels cross-linked with either 6 or 12 mg/ml of 4s-StarPEG and fixed after 48-h incubation period in plating media at 37°C. To limit the impact of sample processing on the cell-encapsulated collagen hydrogels, the samples were dehydrated in ascending ethanol concentrations (50, 70 and 100%) and hexamethyldisilazane and left to air-dry overnight. Samples were mounted and gold sputter-coated. Images were taken using a Hitachi S-4700N Pressure Scanning Electron Microscope (Hitachi, U.K.).

#### Assessment of the collagen hydrogel gelation time

Since the concentration of cross-linker used in the hydrogels will affect the speed and intensity of polymerisation, the gelation time of the collagen hydrogels with increasing concentrations of cross-linker was assessed *in vitro*. Fifty microlitres of collagen hydrogels with increasing cross-linker concentrations (1–12 mg/ml) were transferred to a previously sterilised (UV radiation) superhydrophobic surface (Teflon®) and placed in an incubator at 37°C and 5% CO_2_ and checked every 5–10 min to evaluate their gelation time.

#### Assessment of IL-10 release from collagen hydrogels

The *in vitro* release of IL-10 from the collagen hydrogels was evaluated using a human IL-10 ELISA. In short, 50 ng of human IL-10 was loaded into 50 µl collagen hydrogels with either 2 or 4 mg/ml of cross-linker and left to polymerise at 37°C. Subsequently, each 50 µl collagen hydrogel was incubated in a well of a 24-well plate with SH-SY5Y cells (50000 cells/cm^2^). Human IL-10 containing supernatant was collected at several time points until the collagen hydrogels were fully degraded (not detectable when medium was removed; 4 days after incorporation to the medium). The amount of IL-10 present in the medium was analysed with a human IL-10 ELISA kit (R&D Systems; DuoSet DY217B) following the manufacturer’s protocol.

#### Assessment of the functionality of the IL-10 released from collagen hydrogels

To determine if the IL-10 released from the collagen hydrogels was fully functional, a Poly I:C challenge was used. Embryonic day 14 (E14) primary ventral mesencephalic (VM) cultures (50000 cells/cm^2^) were pretreated with IL-10 (50 ng/ml) either as a single bolus or delivered in a 50 µl collagen hydrogel with 4 mg/ml cross-linker, and 1 h later, Poly I:C (InvivoGen, 20 or 50 µg/ml) was administered. After 24 h of incubation with Poly I:C, medium was collected. Levels of the pro-inflammatory cytokine, IL-1β, released from the VM cultures in response to the inflammagen, Poly I:C, were measured using an ELISA kit (Peprotech; 900-K91) to confirm the functionality of IL-10. Untreated cultures of VM cells, without either Poly I:C, IL-10 or hydrogel treatment, were included as a baseline for IL-1β release.

#### Assessment of the cytocompatibility of the collagen hydrogels

Before using hydrogels *in vivo*, their biocompatibility with VM cultures was assessed. VM cultures were incubated with preformed collagen hydrogels (2 × 50 µl gels) cross-linked with 1, 2 or 4 mg/ml of cross-linker for 24 h or left untreated. Following incubation, cells were either assessed for cell viability using the AlamarBlue® (Invitrogen) assay or fixed for future immunocytochemical staining for neurons (using mouse anti-β-III tubulin from Millipore @ 1:200), dopaminergic neurons (using mouse anti-tyrosine hydroxylase from Millipore @ 1:1000) or astrocytes (using rabbit anti-GFAP from Millipore @ 1:2000, respectively). For analysis, cell counts were quantified from five randomly selected sample sites per well, in three technical replicates per experimental condition, with three biological replicates. The β-III tubulin florescence was quantified by measuring the threshold area of each image using ImageJ software.

### *In vivo* studies

#### Ethical statement and surgical procedures

All procedures involving the use of animals were approved by the Animal Care and Research Ethics Committee at the National University of Ireland, Galway, were completed under licence by the Irish Health Products Regulatory Authority, and were carried out in compliance with the European Union Directive 2010/63/EU and S.I No. 543 of 2012. Male Sprague–Dawley rats (weighing 200–225g on arrival) and time-mated female Sprague–Dawley rats were sourced from Charles River, U.K. Animals were housed in groups of four per cage, on a 12:12-h light/dark cycle, at 19–23°C, with relative humidity levels maintained between 40 and 70%. For the duration of the experiment, animals were allowed food and water *ad libitum*. All *ex vivo* analyses were carried out by an experimenter blind to the treatment of the animals.

All stereotaxic surgeries were performed under isoflurane anaesthesia (5% in O_2_ for induction and 2% in O_2_ for maintenance) in a stereotaxic frame with the nose bar set at −2.3 (intrastriatal) or −4.5 (intra-medial forebrain bundle (MFB)). For infusions to the striatum (*in situ* forming collagen hydrogels), coordinates were AP = 0.0, ML ±3.7 (from bregma) and DV −5.0 below dura, at a total volume of 6 µl per injection. For infusions in the MFB (6-hydroxydopamine lesions), coordinates were AP −4.0, ML −1.3 (from bregma) and DV −7.0 below dura, at a total volume of 3 µl per injection.

To obtain tissue for E14 VM cultures and suspensions, time-mated female Sprague–Dawley rats were deeply anaesthetised with isoflurane (5% in O_2_) and rapidly decapitated using a guillotine. The E14 embryos were obtained by laparotomy and the VM was microdissected from each embryo as previously described [17]. From this tissue, single-cell suspensions were generated for cell culture and intracerebral transplantation as previously described [13,14].

#### *In vivo* study in naïve rats to assess the neurocompatibility of the IL-10-eluting hydrogel as well as its ability to deliver and retain IL-10 in the striatum

To evaluate the effects of cross-linker concentration *in vivo*, 24 adult male Sprague–Dawley rats received a bilateral intrastriatal infusion of 1000 ng (in 6 µl) of human IL-10 loaded into 6 µl of collagen hydrogel cross-linked with either 1, 2 or 4 mg/ml of 4s-StarPEG (*n*=4 per group per time point). A bolus intrastriatal infusion of human IL-10 (1000 ng in 6 µl PBS) was used as a control. The animals were then killed at days 1, 2 or 4 post-surgery by terminal anaesthesia (50 mg/kg pentobarbital i.p.) and transcardial perfusion-fixation for *post-mortem* immunohistochemical analysis of collagen polymerisation and biodegradation, IL-10 delivery and retention, and the host inflammatory response (using rabbit anti-collagen from Abcam @ 1:1000; rabbit anti-IL-10 from Peprotech @ 1:200; mouse anti-CD11b from Millipore @ 1:400; rabbit anti-GFAP from Dako @ 1:2000) as previously described [13,14]. The volume of immunostaining was assessed using cross-sectional areas measured from photomicrographs of a 1:6 series of sections throughout the rostro-caudal axis of the striatum, while for optical density analyses, the staining density was assessed from photomicrographs of three sections along the rostro-caudal axis. To do so, the mean grey values were determined using ImageJ software, and converted into optical density (arbitrary units) by applying the following formula: OD = log10 (255/mean grey value).

#### *In vivo* study in parkinsonian rats to assess the effects of an IL-10-eluting collagen hydrogel on the host inflammatory response to a VM cell transplant

Once the hydrogel neurocompatibility and ability to release IL-10 were determined, an *in vivo* study was carried out to assess if the collagen hydrogel could reduce the host innate immune response after a VM cell transplant in the 6-hydroxydopamine lesioned rat model of Parkinson’s disease [19]. Male Sprague–Dawley rats (*n*=13) received a unilateral intra-MFB 6-hydroxydopamine lesion (12 μg in 3 µl). Rats were then assigned into three groups (*n*=4–5 per group) to receive intrastriatal transplants of E14 VM cells (300000 cells) encapsulated in an IL-10-loaded collagen hydrogel (cross-linked with 4 mg/ml 4s-StarPEG) with either 0, 500 or 1000 ng of IL-10. The animals were killed 4 weeks post-transplantation by terminal anaesthesia (50 mg/kg pentobarbital i.p.) and transcardial perfusion-fixation for *post-mortem* assessment of the host inflammatory response to the transplant (using mouse anti-CD11b from Millipore @ 1:400 and rabbit anti-GFAP from Dako @ 1:2000). Volume and optical density analyses of immunostaining were completed as described above. The 4-week timepoint was chosen as the endpoint of the study as we have previously shown a pronounced host inflammatory response to the transplant as early as 2 weeks after VM cell transplantation [14].

#### Statistical analysis

All data are expressed as mean ± standard error of the mean, and were analysed using one-way, two-way or two-way repeated-measures ANOVA as appropriate, with *post-hoc* Bonferroni test where required. Throughout the ‘Results’ text, the main effects from the initial ANOVA are cited in the body of the ‘Results’ section, while the results of the *post-hoc* analyses are shown in the corresponding figure and explained in the figure legend.

## Results

### *In vitro* characterisation of differentially cross-linked collagen hydrogels

**Figure 3:**
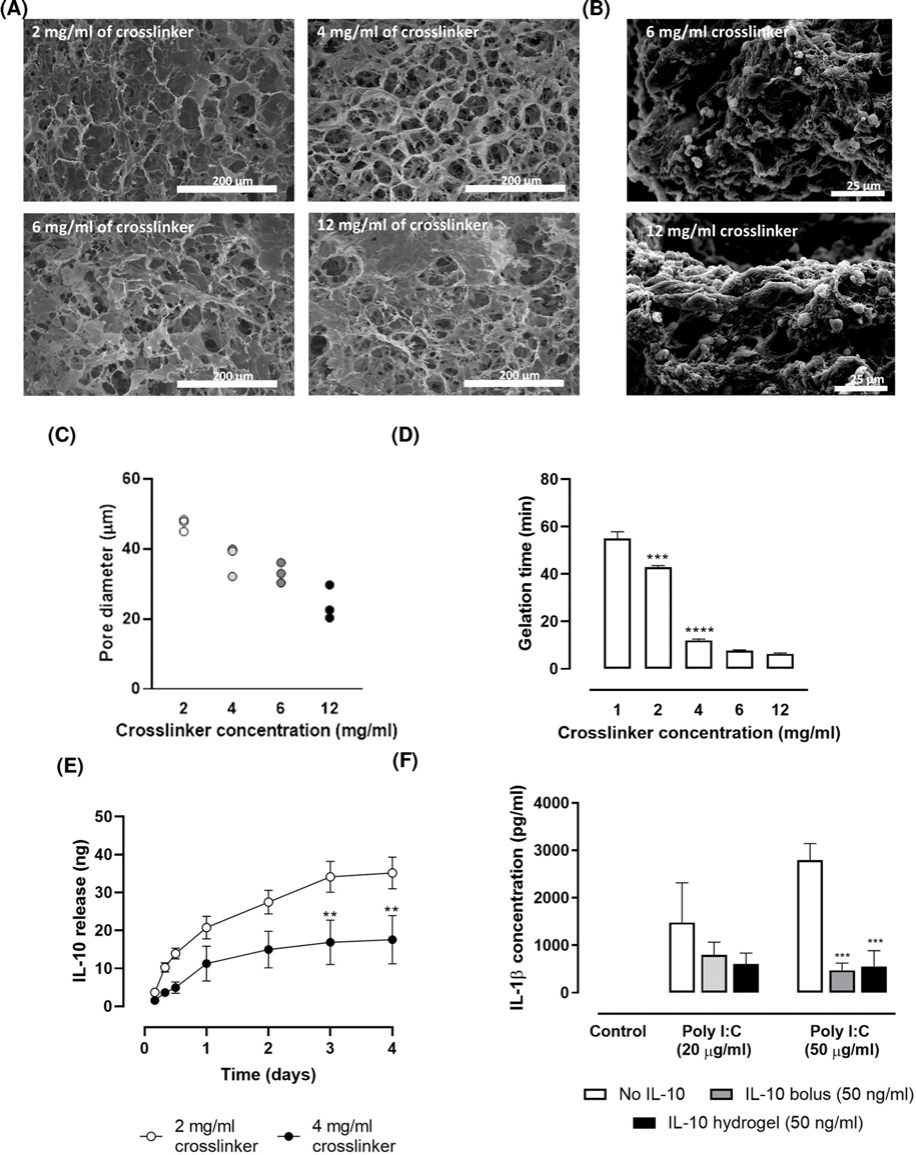
*In vitro* characterisation of the collagen hydrogels (**A**) The pore matrix of the collagen hydrogel was visible under SEM. (**B**) SH-SY5Y cells distributed homogeneously in the collagen cross-linker fibres. (**C,D**) The cross-linking of collagen with increasing levels of 4s-StarPEG reduced the pore size and decreased the time required for gelation. (**E**) Collagen hydrogels with 2 or 4 mg/ml of cross-linker retained and released human IL-10 over time in a neuronal culture. (**F**) The IL-10 released from the collagen hydrogels (4 mg/ml of cross-linker) reduced the pro-inflammatory cytokine levels, IL-1β, in a Poly I:C challenge in primary neural cultures. Data are expressed as mean ± SEM and were analysed by one-way (D) or two-way ANOVA (E,F) with Bonferroni *post-hoc* test. In (D), ***P<0.001, ****P<0.0001 vs previous timepoint. In (E), **P<0.01 vs 2 mg/ml. In (F), ***P<0.001 vs no IL-10 group.

Several collagen hydrogels with a fixed concentration of type I collagen and different 4s-StarPEG concentrations were generated and characterised. The porous structure of the hydrogels was clearly visible using SEM (Figure 3A), allowing for the homogeneous encapsulation of SH-SY5Y cells (Figure 3B). As expected, increasing the cross-linker concentrations reduced the size of the hydrogel pores (Figure 3C). Unsurprisingly, the cross-linker concentration was also strongly linked to the gelation kinetics, with a higher concentration of cross-linker leading to shorter hydrogel gelation times (Figure 3D; cross-linker concentration, *F*_(4,10)_ = 697.60, *P*<0.0001).

To assess the IL-10 elution kinetics, IL-10 was loaded into collagen hydrogels (2 and 4 mg/ml cross-linker), and when fully polymerised, collagen hydrogels were added to SH-SY5Y neuronal cultures. Both collagen hydrogel compositions successfully retained and released IL-10 into the medium over time until fully degraded at 4 days post administration (collagen hydrogels were not visible when medium was removed) (Figure 3E; time, *F*_(6,28)_ = 25.39, *P*<0.0001). The collagen hydrogels with a lower concentration of cross-linker degraded more rapidly, consequently releasing IL-10 into the medium faster (Figure 3E; cross-linker concentration, *F*_(1,28)_ = 57.34, *P*<0.0001).

Furthermore, to ensure IL-10 released from the collagen hydrogels was functional, its effects were assessed using a Poly I:C challenge. The administration of Poly I:C to VM cell cultures generated an inflammatory response as observed by the release of the pro-inflammatory factor IL-1β into the medium (Figure 3F; Poly I:C, *F*_(2,18)_ = 11.84, *P*<0.001). Pretreatment of VM cultures with IL-10 – either as a bolus or released from a collagen hydrogel – 1 h before Poly I:C addition attenuated this response (Figure 3F; IL-10, *F*_(2,18)_ = 93.44, *P*<0.01). Although the kinetics of IL-10 release into the cell culture medium is distinctly different when delivered as a bolus (same dose but higher immediate concentration in the medium) vs from the hydrogel (same dose but slower, more sustained release) the purpose of this assay was simply to confirm that the IL-10 retained its functionality after elution from the gel.

**Figure 4:**
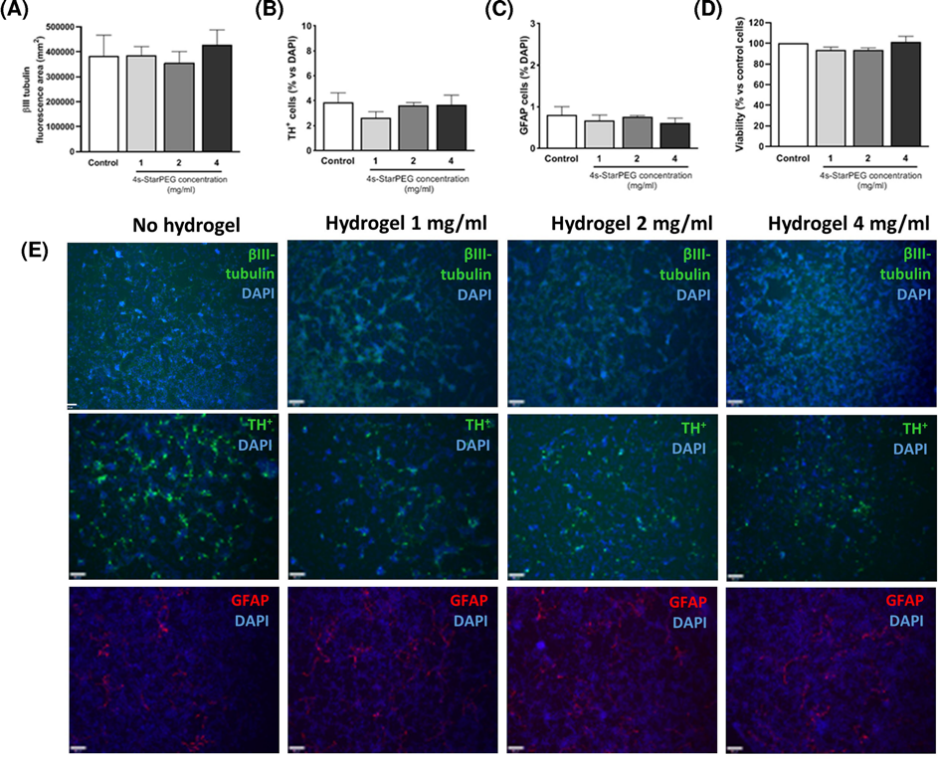
*In vitro* cytocompatibility of the collagen hydrogels As assessed via immunocytochemistry, the incubation of collagen hydrogels with varying 4s-StarPEG concentrations did not alter the mixed populations of the VM cultures such as the (**A**) neuronal population, (**B**) dopaminergic neurons or (**C**) astrocytes, and (**D**) they had no negative impact on the metabolic activity of the VM cultures. Photomicrographs (**E**) represent β-III tubulin, tyrosine hydroxylase and GFAP immunocytochemistry, counterstained with DAPI, respectively. Scale bar represents 100 µm. Data are expressed as mean ± SEM and were analysed by one-way ANOVA with Bonferroni’s *post-hoc* test.

Before conducting *in vivo* experiments, the collagen hydrogel cytocompatibility was assessed by exposing primary VM cultures to preformed collagen hydrogels with increasing concentrations of cross-linker (1–4 mg/ml) for 24 h. All collagen hydrogel compositions were compatible with these primary neural cultures as no change in the populations of neuronal cells (Figure 4A; cross-linker concentration, *F*_(3,8)_ = 0.25, *P>*0.05), dopaminergic neurons (Figure 4B; cross-linker concentration, *F*_(3,8)_ = 0.21, *P>*0.05) or astrocytes (Figure 4C; cross-linker concentration, *F*_(3,8)_ = 0.39, *P>*0.05) were observed. Additionally, the overall viability of VM cultures, as assessed by AlamarBlue® assay, was not compromised by the exposure to any of the collagen hydrogels (Figure 4D; cross-linker concentration *F*_(3,8)_ = 1.41, *P>*0.05). In support of these results, the morphology of neuronal cells, dopaminergic neurons and astrocytes in the VM culture was not modified by any of the tested hydrogel compositions (Figure 4E).

Taken together, these data indicate that the properties of the hydrogel can be tuned by regulating the cross-linker concentration. More importantly, we have shown that collagen hydrogels are cytocompatible, and can retain and release a functional anti-inflammatory cytokine *in vitro.*

### *In vivo* study in naïve rats to assess the neurocompatibility of the IL-10-eluting hydrogel as well as its ability to deliver and retain IL-10 in the striatum

After ensuring that hydrogels were cytocompatible and able to retain and release IL-10 *in vitro*, we assessed these properties *in vivo*. To do so, IL-10-loaded collagen hydrogels with increasing concentrations of cross-linker (1–4 mg/ml) were injected into the striatum of naïve rats.

**Figure 5:**
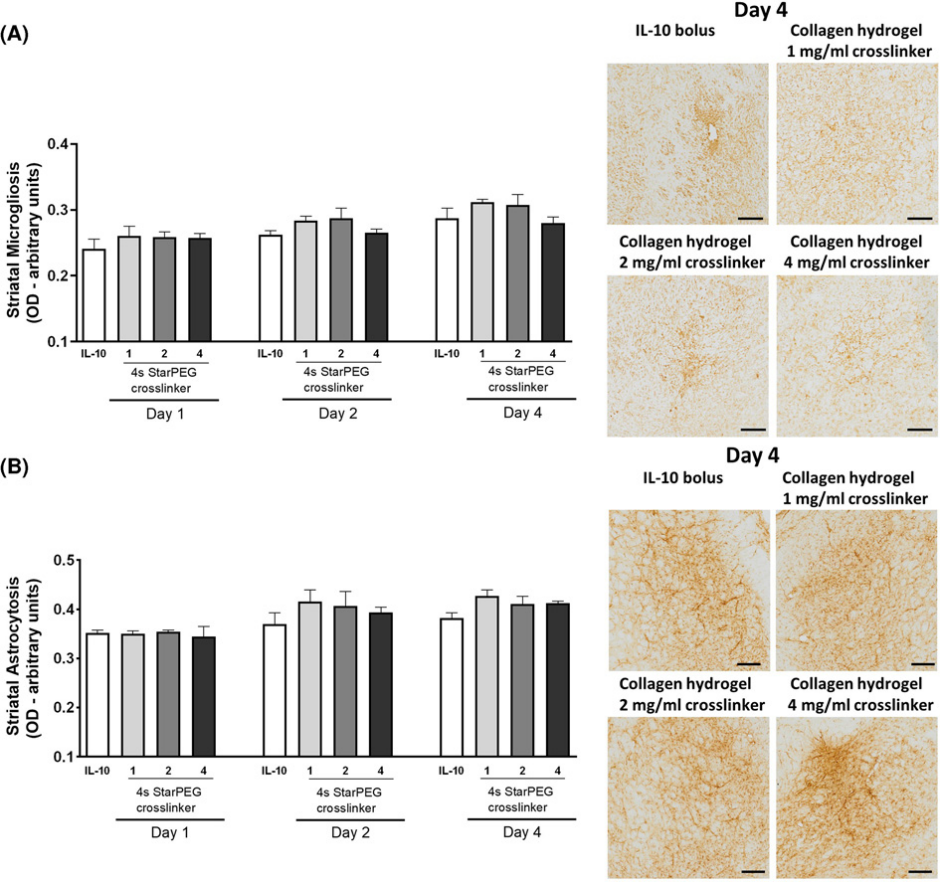
*In vivo* neurocompatibility of the collagen hydrogels IL-10-loaded collagen hydrogels with increasing concentrations of 4s-StarPEG (1–4 mg/ml) were injected into the striatum of naïve rats. Immunohistochemistry analysis showed that the delivery of IL-10 in a collagen hydrogel did not modify the level of either (**A**) microgliosis or (**B**) astrocytosis compared with an IL-10 bolus. Scale bar represents 200 µm. Data are expressed as mean ± SEM and were analysed by two-way ANOVA with *post-hoc* Bonferroni.

We first determined the host neuroinflammatory response to the collagen hydrogels by evaluating the microgliotic and astrocytic reaction in the implant site vicinity (Figure 5). We found that the density of microgliosis (IL-10 delivery, *F*_(3,34)_ = 3.137, *P<*0.05) and astrocytosis (IL-10 delivery, *F*_(3,34)_ = 1.824, *P>*0.05) around the implant site was similar when IL-10 was delivered as a bolus or within the hydrogel. This result suggests that hydrogels cross-linked with 4s-StarPEG at a concentration range of 1–4 mg/ml are biocompatible for intrastriatal delivery of IL-10.

**Figure 6:**
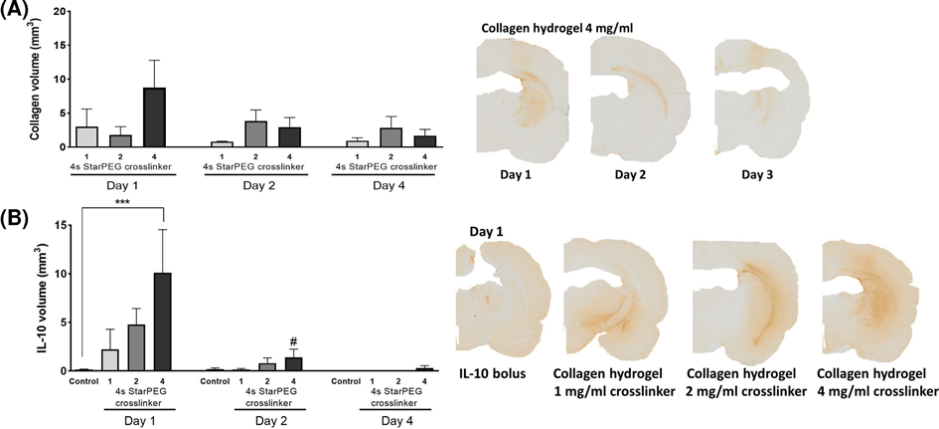
*In vivo* polymerisation and biodegradation of, and IL-10 retention by, the collagen hydrogels Recombinant human IL-10 (1000 ng) was delivered bilaterally to the striatum of naïve rats as a bolus or in a collagen hydrogel (cross-linked with 1–4 mg/ml 4s-StarPEG). (**A**) Collagen staining using immunohistochemistry showed a reduction in collagen staining over time. (**B**) On day 1, the volume of IL-10 was significantly greater when IL-10 was encapsulated in a collagen hydrogel with 4 mg/ml of 4s-StarPEG compared with an IL-10 bolus. Scale bar represents 100 µm. Data are represented as mean ± SEM and were analysed by two-way ANOVA with Bonferroni *post-hoc* test. ***P<0.001 vs IL-10 bolus, ^#^*P*<0.05 vs previous time-point.

Within the same study, type I bovine collagen immunostaining showed the presence of collagen hydrogel in the striatum throughout all experimental groups (Figure 6A) confirming *in situ* polymerisation. The collagen volume was similar between all hydrogel compositions, although the collagen hydrogel with 4 mg/ml of cross-linker showed a greater trend of collagen staining. As expected, the collagen staining tended to decrease with time indicating the biodegradability of the hydrogel.

Once the neurocompatibility, *in situ* polymerisation and biodegradation of hydrogel compositions were verified, the ability of the hydrogels to deliver and retain IL-10 in the surrounding striatum was assessed using IL-10 immunostained photomicrographs. Analysis showed significantly more IL-10 immunostaining in the striatum at 24-h post-implantation when delivered within the hydrogel relative to a bolus injection (Figure 6B; IL-10 delivery, *F*_(3,36)_ = 3.824, *P<*0.05). Indeed, the volume of IL-10 staining increased from 0.14 ± 0.106 mm^3^ to 10.12 ± 7.656 mm^3^, which represents a 70-fold increase in retention of IL-10. Because of the rapid biodegradation of the collagen hydrogel, the volume of IL-10 in the striatum also decreased quickly over time (Figure 6B; time, *F*_(2,36)_ = 9.034, *P<*0.001).

### *In vivo* study in parkinsonian rats to assess the effects of an IL-10-eluting collagen hydrogel on the host inflammatory response to a VM cell transplant

**Figure 7:**
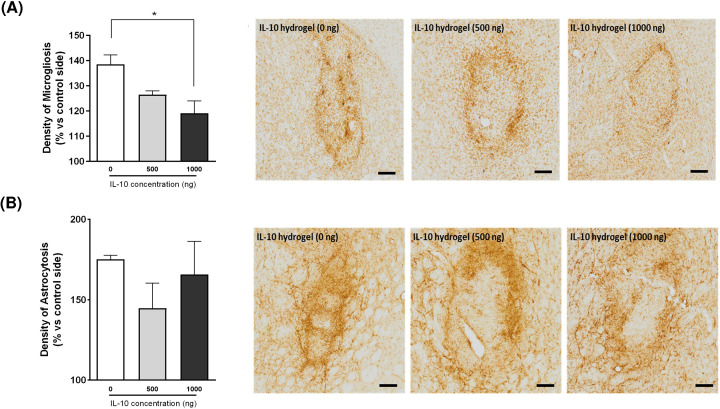
Effects of IL-10-loaded collagen hydrogel on the host inflammatory response to a VM cell transplant Parkinsonian rats (6-hydroxydopamine lesioned) were transplanted with E14 VM cells either in unloaded collagen hydrogels or in collagen hydrogels (4 mg/ml of cross-linker) loaded with IL-10 (500 or 1000 ng). (**A**) The delivery of primary dopaminergic cells in an IL-10-loaded collagen hydrogel resulted in a significant reduction in microglia density at 4 weeks post-transplantation. (**B**) The delivery of VM cells in an IL-10-loaded collagen hydrogel did not change the astrocyte response to the grafted cells. Data are represented as mean ± SEM and were analysed by one-way ANOVA with Bonferroni’s *post-hoc* test. Scale bar = 200 µm. *P<0.05 vs unloaded hydrogel.

In the final study, we assessed whether the IL-10 loaded collagen hydrogels could reduce the inflammatory response elicited by the intrastriatal transplantation of primary dopamine-rich neural transplants. To do so, 6-hydroxydopamine lesioned rats were transplanted with E14 VM cells encapsulated in either unloaded collagen hydrogels, or in collagen hydrogels loaded with IL-10 (500 or 1000 ng). In line with our previous studies, the delivery of the cell-seeded collagen hydrogels with 4 mg/ml of cross-linker did elicit a host innate immune response surrounding the transplantation site (Figure 7). However, the transplantation of VM cells within IL-10 loaded hydrogels (1000 ng dose) significantly reduced the density of microglia surrounding the VM graft (Figure 7A; IL-10 concentration, *F*_(2,10)_ = 6.243, *P<*0.05), showing that the inflammatory response elicited by the intrastriatal transplantation of primary dopamine neuron-rich grafts can be reduced by encapsulation within the IL-10-loaded collagen hydrogel. To note, no collagen or IL-10 immunostaining was detected at this 4 week timepoint indicating that the hydrogels had fully degraded (data not shown).

## Discussion

Because one of the factors that contributes to the varied outcome of cell-based brain repair for Parkinson’s disease is the host brain’s inflammatory response to the implanted cells, in the present study, we sought to generate a collagen hydrogel matrix enriched with the anti-inflammatory cytokine, IL-10, for reduction in this neuroinflammatory response. In the first instance, we generated several hydrogel compositions using different concentrations of cross-linker and assessed these *in vitro* for their microstructure and gelation kinetics, as well as their IL-10 elution kinetics, anti-inflammatory functionality and cytocompatibility. After this initial characterisation, we assessed the IL-10-eluting hydrogels *in vivo* to deliver and retain IL-10 within the striatum, and their ability to reduce the neuroinflammatory response to dopaminergic grafts.

*In vitro* studies revealed that increasing cross-linker concentration reduced the hydrogels’ pore size and accelerated their gelation kinetics, but did not affect the cytocompatibility of the hydrogels. Most importantly, the *in vitro* studies demonstrated that hydrogels could release IL-10 over time and that the released cytokine retained its anti-inflammatory functionality. Subsequently, the *in vivo* studies demonstrated the ability of the IL-10-loaded collagen hydrogels to deliver and retain the cytokine within the striatum (relative to bolus injection of the cytokine), and most importantly in the context of cell-based brain repair for Parkinson’s disease, to reduce the microglial neuroinflammatory response to dopaminergic grafts. Taken together, these data suggest that anti-inflammatory functionalised hydrogels merit further exploration as a delivery matrix for cellular reparative strategies in Parkinson’s disease.

Biomaterials, particularly injectable hydrogels, have the potential to improve the outcome of brain repair strategies for Parkinson’s disease through multiple mechanisms. Several studies have shown that the intrastriatal transplantation of dopaminergic grafts encapsulated in collagen hydrogels can provide the transplanted cells with a more favourable microenvironment during and after transplantation that ultimately improves the efficacy of this approach [13,14]. One mechanism through which hydrogels function is by providing a physical barrier between the exogenous transplanted cells and the host brain’s innate inflammatory cells thereby dramatically reducing the recruitment of astrocytes and microglia around the graft site [14,15]. Several studies have focused on enhancing the neurotrophic properties of the hydrogels through enrichment with dopaminergic neurotrophins such as GDNF [13,14]. Still, to date, no study has focused on enhancing its anti-inflammatory properties. Thus, in the present study, we sought to generate a collagen hydrogel enriched with the anti-inflammatory cytokine, IL-10, to further enhance the favourable microenvironment provided by the hydrogel to cells encapsulated within it.

In the studies presented here, we have used collagen hydrogels fabricated with bovine type I collagen and multiple concentrations of 4s-StarPEG (1–12 mg/ml) as the main components of the biomaterial as they have been successfully used for cell transplantation previously [13,14]. Type I collagen was chosen since it has been widely used for cell encapsulation due to its properties of mimicking the extracellular matrix and its ability to polymerise and form an *in situ* hydrogel [12], whereas 4s-StarPEG can stabilise and stiffen the resulting collagen hydrogel while being non-toxic, with low immunogenicity and already approved by the U.S. Food and Drug Administration [18]. The concentration of collagen in the collagen hydrogels was not investigated in this work as collagen hydrogels with 2 mg/ml of type I bovine collagen have successfully been used in cell transplantation studies for Parkinson’s disease [13,14].

In the initial studies, as the hydrogel structure can be regulated by the cross-linker agent [18], we analysed several collagen hydrogel compositions (with increasing concentration of cross-linker) to determine how the concentration of 4s-StarPEG cross-linker altered its properties. The degree of cross-linking is a crucial component for many of the hydrogel’s properties like elasticity, rigidity and swelling behaviour. Here, we have shown that the cross-linker concentration did modify the fibrous microstructure of the collagen hydrogel. Furthermore, we showed that higher the cross-linker concentration, the quicker the polymerisation occurred *in vitro* at 37°C. We have also demonstrated here that the hydrogels could release IL-10 over time in *in vitro* neuronal cultures, until the hydrogel was fully degraded. More importantly, we have shown by using a Poly I:C challenge that the released IL-10 can exert its biological functions such as reducing the levels of the pro-inflammatory cytokine IL-1β. Since chemical cross-linking can result in toxicity, the compatibility of the biomaterial must be assessed *in vitro*, before using the hydrogels in the brain. Our preliminary *in vitro* assessments showed that the incubation of hydrogels along with VM cultures did not have any negative impact on the cell survival at any of the cross-linker concentrations assessed. Together the *in vitro* data suggested that collagen hydrogels with lower concentrations of cross-linker (1–4 mg/ml of 4s-StarPEG) may be the best candidates for *in vivo* delivery since they were cytocompatible, had slow gelling properties and were able to retain and release functional IL-10.

In our first *in vivo* study, all collagen hydrogel compositions (with 1–4 mg/ml of cross-linker) injected into the striatum successfully polymerised *in situ* and the collagen hydrogel with the greater cross–linker concentration (4 mg/ml) showed the strongest and most defined collagen staining at 24 h post-transplantation. The collagen staining was less well-defined for the 1 and 2 mg/ml cross-linked hydrogels, probably reflecting poor *in situ* formation and/or rapid biodegradation. As expected, the 4 mg/ml collagen hydrogel degraded quickly over time as it was mostly degraded by day 4 post-injection as seen previously [14]. Similar to other type I collagen hydrogels injected into the brain [13–15], we determined that the collagen hydrogels were compatible with the host striatum considering the host immune response generated by microglia and astrocytes was comparable with an IL-10 bolus injection.

However, the most striking finding of our first *in vivo* study was the extent to which the collagen hydrogel, with 4 mg/ml of cross-linker, retained IL-10 in the striatum relative to a bolus injection at 24 h post-implantation. Although the retention time of IL-10 was short, it is well established that the immediate post-grafting window is the most critical for dopaminergic cell transplants [19,20] and that the host inflammatory response to the cells manifests within hours after transplantation [3–5,7–10]. This suggests that the increased retention of this anti-inflammatory cytokine, even for short periods, could have beneficial consequences for grafting. Indeed, we have already reported significant benefits from retaining trophic factors for short periods alongside transplanted cells. For example, in the Moriarty et al. studies [13,14], even though GDNF was fully degraded by day 4 post-injection, it dramatically improved the survival of primary dopaminergic cells.

Having established that the IL-10-eluting hydrogels were well tolerated in the brain and capable of IL-10 retention in the striatum, we sought to assess if they could reduce the inevitable host inflammatory response to intrastriatal dopamine neuron-rich grafts. Although the delivery of cells in collagen scaffolds for neural repair has been investigated, this is the first time that cells have been delivered in an anti-inflammatory cytokine enriched collagen scaffold. In this work, we have shown that the encapsulation of VM cells in an IL-10-loaded collagen hydrogel reduced the density of microglial cells around the graft site at 4 weeks post-transplantation. The suppression of microglial activation by IL-10 is well established [16] but this is the first time it has been shown in the context of brain repair for Parkinson’s disease. Ultimately, further long-term studies will be required to determine if this anti-inflammatory effect provides additional benefit to the encapsulated cells and further improves the efficacy of this approach.

In conclusion, we found that collagen hydrogels enriched with the anti-inflammatory cytokine, IL-10, were highly cytocompatible *in vitro* and neurocompatible *in vivo*, and could release functional IL-10 both in cellular models and in the Parkinsonian brain where it reduced the microglial response to dopaminergic grafts. Taken together, these data suggest that anti-inflammatory functionalised hydrogels merit further exploration as a delivery matrix for cellular reparative strategies in Parkinson’s disease.

## Data Availability

The data that support the findings of the present study are available from the corresponding author, upon reasonable request.

## Abbreviations

AP: anterior-posterior
DV: dorsal-ventral
E14: embryonic day 14
GDNF: glial cell line-derived neurotrophic factor
IL: interleukin
i.p: intraperitoneal
MFB: medial forebrain bundle
ML: medial-lateral
PBS: phosphate-buffered saline
SEM: scanning electron microscopy
VM: ventral mesencephalic
4s-StarPEG: poly(ethylene glycol) ether tetrasuccinimidyl glutarate
